# Use of Fantasy Points in Evaluating Professional Athlete Performance After Anterior Cruciate Ligament (ACL) Reconstruction

**DOI:** 10.7759/cureus.35855

**Published:** 2023-03-07

**Authors:** Marvin Kajy, Devan O Higginbotham, Ali Etemad-Rezaie, Guy R S Ball, Rahul Vaidya

**Affiliations:** 1 Cardiovascular Medicine, Spectrum Health, Grand Rapids, USA; 2 Orthopaedic Surgery, Wayne State University Detroit Medical Center, Detroit, USA; 3 Orthopaedic Surgery, University of Toronto, Toronto, CAN; 4 Orthopaedic Surgery, McLaren Oakland Hospital, Pontiac, USA

**Keywords:** acl, sports rehabilitation, return to play, fantasy points, professional athletes

## Abstract

Our aim in performing this study was to evaluate whether fantasy and wins above replacement (WAR) scores of athletes undergoing anterior cruciate ligament (ACL) reconstructive surgery in the National Football League (NFL), National Basketball Association (NBA), National Hockey League (NHL), and Major League Baseball (MLB) could be utilized in evaluating their performance post-surgery. We identified publicly accessible data on professional athletes from 1992 to 2015. Fantasy and WAR scores were calculated for each player. A total of 83 professional players met the inclusion criteria for this cross-sectional study. Decreased fantasy scores ranged from 33% to 42% across the four leagues after the index operation. NHL players had the lowest return-to-play (RTP) rate at 11/17 (82%), and MLB players had the highest RTP rate at 14/15 (93%). RTP rates of NBA and NFL players were comparable at 22/26 (85%) and 22/25 (88%), respectively. NFL players had the lowest average career length after surgery at 26 months, while NBA players had the longest average career length at 64 months. MLB players on average required the longest time to return to the pre-surgical level of performance (21 months). NHL players had the shortest average recovery time (eight months), and NBA players had the longest average recovery time (13 months). Approximately, more than half of all the studied players exhibited a decline in fantasy or WAR scores. In addition, NFL players had the lowest average career length, and NBA players enjoyed the longest average career length after surgery. NHL players had the lowest recovery time, while NBA players had the longest recovery time. The strength of this study is the utilization of fantasy points and WAR scores as a single unifying measure of a player’s performance, which acts as an objective measure after ACL reconstruction. The average performance of a professional athlete, as evaluated through their fantasy score output, tends to decrease after undergoing ACL reconstruction. There is an overall long-term performance decline after initial spikes in their performance after surgery. Additional larger studies are needed to fully understand the effects of ACL reconstruction in professional athletes; however, the use of fantasy scores may be an objective tool in measuring the success rate of ACL reconstruction.

## Introduction

The involvement in fantasy sports has seen a dramatic rise in interest since its inception in 1979 [1,2]. Fantasy sports has emerged as a billion-dollar industry in the United States alone and is estimated to be worth approximately $22.31 billion globally in 2021 at a compound annual growth rate (CAGR) of 9.5% [3]. Player performance can be measured using fantasy scores, and a decline in performance may be an indication of injury or a decline in the overall peak physical form. Anterior cruciate ligament (ACL) injuries are a prevalent concern not only for players themselves but also for any lay individual who may involve a player in one of their “leagues.” The goal of ACL reconstruction is to successfully restore the native anatomy and biomechanical function of the native ligament, provide a stable and pain-free knee joint, allow return to the prior level of activity, prevent re-rupture, and prevent osteoarthritis [4].

Previous studies have shown a return-to-play (RTP) rate of players undergoing ACL reconstruction ranging from 63% to 93% across the professional leagues of Major League Baseball (MLB), National Basketball Association (NBA), National Hockey League (NHL), and National Football League (NFL) [4-7]. Ardern et al. [5] revealed that elite athletes have greater odds of returning to any sport after surgery; however, the rates of return to the pre-injury level and competitive level sport remain low [4]. Fabricant et al. [8] showed that 88% of MLB players were able to return to sport after surgery, but they did not address career length, recovery time, or return to pre-injury level [8]. An analysis of NHL players by Erickson et al. concluded that athlete performance following ACL reconstruction did not differ significantly from preinjury by evaluating time played, scored goals, shooting percentage, and shooting attempts [9]. Shah et al. [10] studied NFL players and reported that the RTP rate of NFL players was 63% at an average of 10.8 months after surgery. Busfield et al. [11] addressed the performance outcomes of ACL reconstruction in NBA players and reported that 22% of players did not return to professional play and noted a decrease in performance with approximately 50% of players who did return to sport. Despite all these studies, data regarding ACL reconstruction in elite athletes remain limited [12].

The objective of this study was to evaluate the effect of ACL reconstruction surgery on player performance in a cohort of players in the NFL, NBA, NHL, and MLB. Our aim in performing this study was to evaluate whether fantasy and wins above replacement (WAR) scores of athletes undergoing ACL reconstructive surgery in NFL, NBA, NHL, and MLB players could be utilized in evaluating their performance post-surgery. In addition, we examined RTP, average career length, average length of time to return to pre-surgical performance, and recovery time. We hypothesized that professional athletes would exhibit an overall decrease in performance after undergoing ACL reconstruction surgery, and we wanted to explore whether fantasy and WAR scores could be an objective means to evaluate player performance after surgery.

## Materials and methods

In the first phase of this study, players in the NFL, NBA, NHL, and MLB who underwent ACL reconstruction were identified through various publicly accessible data from 1992 to 2015. The main sources of information were obtained from ESPN (http://espn.go.com), NFL (http://www.nfl.com), NBA (http://www.nba.com), NHL (http://www.nhl.com), and MLB (http://www.mlb.com). The inclusion criteria involved players missing at least one game during the regular or postseason due to ACL reconstruction surgery. In the second phase of the study, players who underwent more than one ACL reconstructive surgery were excluded from this study. Player information such as position, number of games played, fantasy points, and WAR scores were compiled. For all leagues other than the MLB, this information was obtained from Rotoworld (http://www.rotoworld.com). MLB player information was collected from FanGraphs (http://www.fangraphs.com). This was in accordance with a previously established methodology [13].

Other parameters examined were time to return to play, percentage of players that were able to achieve pre-injury performance, career length after the index operation, length of time to return to the pre-surgical level of performance, and recovery time. In this study, the RTP rate was defined as the percentage of athletes returning to professional play after undergoing the procedure. Career length after the index operation was defined as the number of years played after undergoing surgery. Recall that the time interval of this study was from 1992 to 2015. There are players who continued to play after 2015, and therefore, the reported career length is an underestimate. The length of time to return to the pre-surgical level of performance was defined as the time taken by the player to reach a fantasy value or WAR after surgery, which is equivalent to the score the year before surgery. Recovery time was defined as the length of time between surgery and the return to competitive play in the patients' sports.

The fantasy scores for NFL players are calculated from a sum. To illustrate, each action that a player performs is worth a specific point. At the end of the season, the sum yields a fantasy score for the player [13]. 

Next, the fantasy points in the NHL [13] are calculated using formulas based on a player’s position. Specifically, the formula for defensemen is:

Fantasy Score = Penalty Minutes/4 + 5*Goals + 4*Assists + 2*Power play Goals + 3*Game Winning Goals + 1*Plus/Minus + 3*Shorthanded Goals

The formula for forwards is:

Fantasy Score = Penalty Minutes/4 + 4*Goals + 3*Assists + 2*Power play Goals + 3*Game Winning Goals + 1*Plus/Minus + 3*Shorthanded Goals

The formula for goalies is:

Fantasy Score = 2*Wins + 1*Overtime Losses + 1*Goals + 1*Assists + 2*Shutouts + Saves/5 - 1*Losses - .75*Goals Allowed

Moreover, the fantasy points for NBA players are not calculated using standard points but are based on the league. A standard league consists of nine categories, including turnovers and 12 teams. The nine-category league fantasy scores are calculated using the following: (1) points scored by the player, (2) field goal percentage, (3) free throw percentage, (4) three-point field goals, (5) assists, (6) steals, (7) blocks, (8) rebounds, and (9) turnovers [13].

To track the performance of MLB players [13], WAR is used instead of fantasy points. WAR values for each MLB player were obtained from FantasyGraphs on the FanGraphs website. WAR is one statistic that assesses a player’s contribution to the team. WAR utilizes various inputs, which is illustrated by the below formula for position players:

WAR = (Batting Runs + Base Running Runs + Fielding Runs + Positional Adjustment + League Adjustment +Replacement Runs) / (Runs Per Win)

## Results

**Figure 1:**
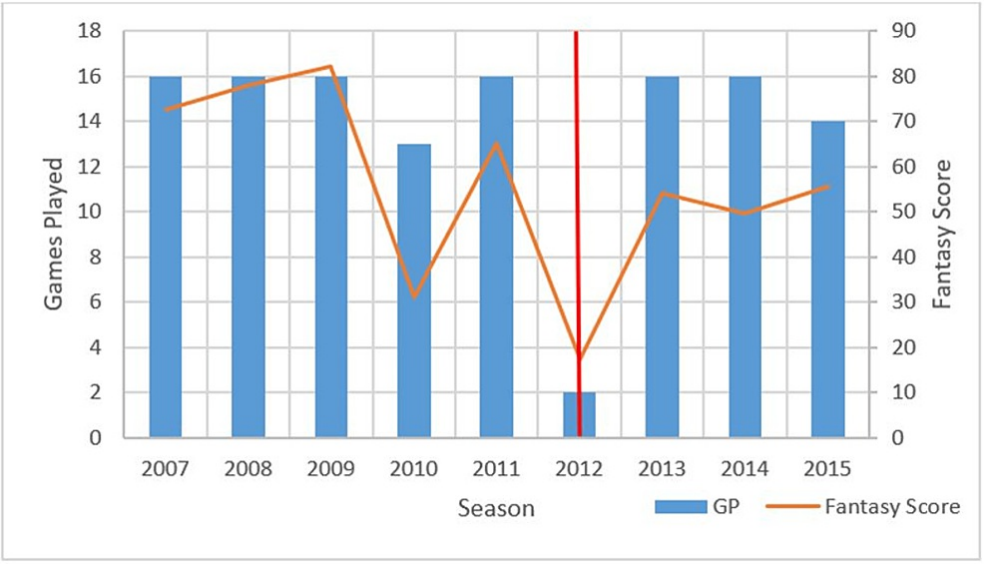
Number of games played (GP) and fantasy scores of an example National Football League (NFL) player over each season. The red line indicates the year of the index operation.

A total of 83 players met the inclusion criteria of this study. These players were split into cohorts depending on which league they participated (NFL, NBA, NHL, and MLB). For each player, the number of games played and fantasy scores for each season were tabulated and graphed as illustrated in Table 1 and Figure 1, respectively.

**Table 1:** Table outlining the number of games played (GP) and fantasy scores with respect to each season of an example National Football League (NFL) linebacker. The year of the index operation is 2012.

Season	2007	2008	2009	2010	2011	2012	2013	2014	2015
GP	16	16	16	13	16	2	16	16	14
Fantasy score	72.5	78	82	31	65	17.5	54	49.5	55.5

The year of the surgery is marked by a red vertical line. Note that for this player, there is an upward trend in the number of games played and fantasy scores after the operation. The average number of games per year post-operation was higher compared to games played pre-operation. However, the player’s average fantasy score per year slightly decreased after the operation.

We examined the performance of 25 NFL players. Table 2 illustrates the outcomes of ACL reconstructive surgery in NFL players. Thirteen players (50%) exhibited a decrease in fantasy scores after undergoing ACL surgery, while six players (23%) displayed an increase in fantasy scores after surgery. Interestingly, 15 players (58%) showed an increase in the number of games played per season.

**Table 2:** Distribution of how players performed after ACL reconstruction surgery in the NFL (n=25). For example, six players exhibited an increase in the number of games played and fantasy scores after surgery. However, three players stopped playing after surgery ACL: anterior cruciate ligament; NFL: National Football League

Fantasy Score	Change in Number of Games Played Season After Index Surgery			
	Increased	Decreased	Unchanged	Stopped
Increased score	6			
Decreased score	7	4	2	
Unchanged score	2	1		
Stop				3

Next, we looked at the statistics of 26 NBA players. Table 3 demonstrates the outcomes of ACL reconstructive surgery in NBA players. A total of 14 players (54%) demonstrated a decrease in fantasy value after the index operation. In addition, 12 players (46%) saw an increase in the number of games played, while six players (23%) played fewer games per season.

**Table 3:** Distribution of how players performed after ACL reconstruction surgery in the NBA (n = 26) ACL: anterior cruciate ligament; NBA: National Basketball Association

Fantasy Score	Change in Number of Games Played Season After Index Surgery			
	Increased	Decreased	Unchanged	Stopped
Increased score	6		2	
Decreased score	6	6	2	
Unchanged score				
Stop				4

We examined the statistics of 17 NHL players. Table 4 demonstrates the outcomes of ACL reconstructive surgery in NHL players. Eight players (47%) showed a decrease in fantasy value status post ACL reconstruction surgery. Three players (17%) played fewer games per season, with three players (17%) not returning to play. Lastly, we looked at the statistics of 15 MLB players (see Table 5). Nine players (60%) exhibited a decrease in WAR. Additionally, seven players (47%) played more games per season after having the surgery.

**Table 4:** Distribution of how players performed after ACL reconstruction surgery in the NHL (n=17) ACL: anterior cruciate ligament; NHL: National Hockey League

Fantasy Score	Change in Number of Games Played Season After Index Surgery			
	Increased	Decreased	Unchanged	Stopped
Increased score	1	1		
Decreased score	2	3	3	
Unchanged score			1	3
Stop				3

**Table 5:** Distribution of how players performed after ACL reconstruction surgery in the MLB (n=15) WAR: wins above replacement; ACL: anterior cruciate ligament; MLB: Major League Baseball

WAR	Change in Number of Games Played Season After Index Surgery			
	Increased	Decreased	Unchanged	Stopped
Increased score	3	1		
Decreased score	3	6		
Unchanged score	1			
Stop				1

Table 6 illustrates a comparison of surgical outcomes between players in terms of RTP, percentage of players that were able to return to pre-injury performance, career length after the index operation, time to return to the pre-surgical level of performance, and recovery time between the four leagues.

**Table 6:** Comparison of RTP, percentage of players that were able to return to pre-injury performance, career length after the index operation, time to return to the pre-surgical level of performance, and recovery time between the four leagues. RTP: return to play

	NFL	NBA	NHL	MLB
RTP	88	85	82	93
Percentage of players that were able to reach pre-injury performance	36	42	35	33
Average length of time to return to the pre-surgical level of performance (months)	15	18	14	21
Average career length after the index operation (months)	26	64	37	42
Average recovery time (months)	11	13	8	9

One can see that NHL players had the lowest RTP rate (82%). The RTP rate in the NFL, NBA, and MLB are comparable. Interestingly, it appears that across the four leagues, approximately 30-40% of players were able to reach pre-injury performance after surgery. However, MLB players on average required the longest time to reach that level (21 months). NFL players had the lowest average career length after surgery (26 months), while NBA players had the longest career length (64 months). The average recovery time between the four leagues is comparable, ranging from 9 to 13 months.

## Discussion

A total of 83 players with confirmed ACL reconstruction surgery and missed time were included in this analysis. The ACL reconstruction surgery is very successful as is illustrated by the high RTP. Interestingly, surgery had a relatively comparable effect on performance across the four leagues. Approximately, half of all the players who were studied exhibited a decline in fantasy or WAR scores in the case of baseball athletes. This indicates that while the RTP remains high at around 83% [14], players do experience a decline in their athletic performance. This decline in athletic performance remained consistent and did not normalize over time. There could be multiple factors contributing to the athlete’s performance decline such as aging, other injuries, and psychological factors; however, the presence of a similar decline across four major professional sports leagues points to the ACL injury as the major contributing factor. As a result, it is important for the medical team to counsel professional athletes about the possible impact of the injury on future performance levels.

When compared across different professional sports leagues, NFL players had the lowest average career length, which could be attributed to the extreme physical demands of the sport [15]. Interestingly, NBA players enjoyed the longest average career length after surgery. This is surprising because ACL injury tends to occur more commonly in sports that have rapid jumping, pivoting, and cutting maneuvers. Such maneuvers are very common in the game of basketball. The RTP and career longevity after significant injury at the elite level can be influenced by multiple factors. Some players may have sustained other injuries during play or in rehab that include but are not limited to their ACL injuries, which can impair them from reaching their pre-injury level. Other causes could be personal, financial, and/or psychological reasons [16]. NHL players had the lowest recovery time, while NBA players had the longest recovery time. This may be a reflection of the relative physical demands of each sport and differences in medical adherence protocol or differences in medical clearance protocols. The game of basketball has maneuvers that may predispose the player to ACL injury such as a player’s increased tendency to drive toward the basket, cut, and pivot [17]. This can influence the recovery time and explain the prolonged rehabilitation prior to full RTP.

Our study contributes to a growing body of literature that seeks to explain the functional outcomes of an elite athlete status post-ACL reconstruction. The strength of this study is the utilization of fantasy points and WAR scores as a single unifying measure of a player’s performance. These values allow one to track how the player performs over time, and they also reveal the magnitude of the change in performance. In addition, all the data used to perform the study came from official websites that tabulate players’ statistics. The last strength of this study is the large number of players studied who play in the four most popular sports leagues in North America.

Study limitations

Our study results are subject to several limitations. First, our small sample of male-only professional athletes may not comprise a true representation of all types of elite athletes. Another limitation concerns the inherent variations of reporting sources of performance-based outcomes and complications that may have skewed our outcome measures. Fantasy scores of certain position players have the potential to be substantially different based upon the inherent nature of fantasy score reporting and may have skewed our results regarding average performance. Our study lacked age-matched controls, which may have provided a more rigorous longitudinal performance representation of aging athletes over time. We do acknowledge that age was not a component of comparison in our studies, which may naturally provide a decline in performance as professional athletes progress in their careers and get closer to retirement. A final limitation concerns the use of statistics websites and reliance on publicly accessible news information sources prone to reporting bias. The detailed medical information of each athlete was limited to medical personnel documentation, and data regarding some complications or patient-specific factors that could have confounded our interpretation of available data were not always available. Some players thus may have suffered additional injuries that were not disclosed, which may have impacted their performance post-surgery. Additionally, information regarding graft selection, the presence of concomitant knee injury, and the surgical technique utilized in each athlete were not made widely available through our means of data collection; thus, any differences related to graft selection were not quantifiable in our study.

## Conclusions

The average performance of professional athletes tends to decline after undergoing ACL reconstruction based upon a player’s fantasy score performance. Performance peaks in the initial years after the operation for some players; however, there exists an overall long-term performance decline. More studies are needed to better understand the effects of ACL reconstruction regarding professional athletes; however, the use of fantasy scores may be an objective tool in measuring the success rate of ACL reconstruction in this population.
